# Scoping Review of Occupational Therapy for Distal Radius Fractures, Including Complications

**DOI:** 10.1155/oti/9148924

**Published:** 2026-07-16

**Authors:** Yuki Inoue, Junichiro Muranaka, Yuko Shigeta, Kohei Ikeda, Masatoshi Gocho, Satoshi Sasada

**Affiliations:** ^1^ Doctoral course of Health and Social Services, Kanagawa University of Human Services Graduate School, Kawasaki, Japan; ^2^ Department of Rehabilitation, Haruyama Memorial Hospital, Shinjuku, Japan; ^3^ Department of Occupational Therapy, Chigasaki Rehabilitation College, Chigasaki, Japan; ^4^ Department of Rehabilitation Sciences, Faculty of Health Sciences, Div. Occupational Therapy, University of Tokyo Health Sciences, Tama, Japan; ^5^ Department of Health & Social Work, School of Rehabilitation, Div. Occupational Therapy, Kanagawa University of Human Services, Yokosuka, Japan, kuhs.ac.jp; ^6^ Department of Occupational Therapy, School of Health Sciences at Odawara, International University of Health and Welfare, Odawara, Japan, iuhw.ac.jp; ^7^ Department of Health and Social Services, Kanagawa University of Human Services Graduate School, Kawasaki, Japan

**Keywords:** complication, distal radius fracture, interventions, outcomes, review

## Abstract

This scoping review is aimed at identifying and mapping occupational therapy–related assessments and interventions reported in prospective interventional studies and case reports on distal radius fractures, including studies addressing complications. Additionally, the review sought to summarize the current evidence in this field and identify areas requiring further research. Five studies met the inclusion criteria. Most studies primarily emphasized impairment‐level outcomes, such as range of motion and grip strength, whereas activity, participation, occupational performance, and psychosocial aspects were addressed less frequently. Only a limited number of studies reported complications, and the included studies generally employed relatively narrow eligibility criteria. These findings suggest that existing evidence may have limited applicability to the broader population of patients with distal radius fractures, particularly those with complications. Future research should include broader patient populations and incorporate occupation‐focused approaches to better address activity, participation, and occupational performance in distal radius fracture rehabilitation.

## 1. Introduction

Distal radius fractures (DRFs) are one of the most common upper‐extremity fractures across all age groups, and their burden is increasing in some populations, although regional variations exist [1–3]. A DRF is generally defined as a fracture occurring at the distal end of the radius near the wrist. DRFs demonstrate a bimodal age distribution, occurring in younger individuals after high‐energy trauma and in older adults, particularly postmenopausal women, after low‐energy trauma such as falls [1]. Epidemiological studies in Europe and the United Kingdom have consistently shown that DRFs are more common in women and are associated with increasing age. Candela et al. reported that DRFs accounted for 6.9% of adult fractures in a large suburban population, while Stirling et al. identified DRFs as the most common adult orthopedic fracture and predicted a 23% increase in fracture incidence in the United Kingdom over the next 20 years [2, 3]. In the United States, this type of fracture is particularly common [4], and its incidence is expected to increase with age [5]. The importance of early intervention in occupational therapy for DRFs lies in restoring the patient′s ability to perform daily activities and reducing physical dysfunction [6]. Because DRFs can affect not only wrist and hand functions but also self‐care, domestic activities, work, leisure, and social participation, occupational therapists are well positioned to support recovery by addressing the relationships among body functions, activity, participation, and the individual′s occupational context.

Notably, DRFs can lead to several complications, including triangular fibrocartilage complex (TFCC) injuries, complex regional pain syndrome (CRPS), malunion, infection, and finger flexor or extensor tendon complications [7, 8]. TFCC injuries reportedly affect 45.8% of patients with DRFs, and the incidence of CRPS ranges from 1% to 51% [7, 9]. Pain may persist for up to 1 year after a DRF, and associated ulnar styloid fractures may adversely affect wrist functional outcomes [1, 10, 11]. Therefore, patients with pain and associated complications following DRF tend to experience a prolonged clinical course. In addition to physical dysfunction, patients with DRFs may experience psychosocial difficulties, including pain‐related fear, anxiety, reduced confidence in using the affected hand, emotional distress, and limitations in resuming daily roles and occupations [12, 13]. These psychosocial and occupational consequences are clinically important because psychological factors, social context, and difficulties in activity and participation may influence recovery, occupational performance, and patient‐reported outcomes after DRFs [13–15]. However, daily activities and occupations are reported to play a substantial role in facilitating functional recovery in patients with DRFs, indicating the importance of interventions that focus not only on functional training but also on occupations embedded in everyday life [16].

From this perspective, occupation‐focused approaches are particularly relevant in DRF rehabilitation. In this review, occupation‐focused approaches refer to assessments and interventions that explicitly address activity, participation, occupational performance, and client‐identified occupational goals, rather than focusing solely on impairment‐level outcomes such as range of motion, grip strength, pain, or edema. The Canadian Occupational Performance Measure (COPM) is an example of an occupation‐focused assessment that evaluates clients′ perceived occupational performance and satisfaction [17]. In addition, the International Classification of Functioning, Disability and Health (ICF) provides a useful framework for understanding recovery after DRFs in terms of body functions and structures, activity, participation, and contextual factors and has been used to analyze occupational performance 1 year after DRFs [13].

Trzeciak and Małek reported that patients with complications are typically excluded from participant selection in occupational therapy interventional studies on DRFs, which limits the participant population [18]. Although several studies have examined specific occupational therapy interventions for DRFs, existing research is fragmented and often focuses on selected patient populations or isolated outcomes. Moreover, because DRF rehabilitation is frequently provided within hand therapy contexts, where occupational therapists and physical therapists may have overlapping roles, it is important to clarify how occupational therapy–related assessments and interventions have been reported in the literature. Previous studies on occupational therapists′ perspectives in hand therapy suggest that practice in this field has historically been influenced by biomechanical approaches and that integrating occupation‐focused assessments and interventions remains an ongoing challenge [19–21]. To date, no comprehensive synthesis has mapped occupational therapy assessments and interventions for DRFs while explicitly considering the inclusion of patients with complications.

Therefore, the objectives of this study are as follows: (1) to identify and map how occupational therapy–related assessments and interventions have been reported in prospective interventional studies and case reports on DRFs, including studies addressing complications, and (2) to summarize occupational therapy research in this field and identify factors requiring future studies. A scoping review methodology was used to conduct the literature review [22, 23]. Using this method enabled us to identify research on occupational therapy involving patients with DRFs, regardless of complications.

## 2. Materials and Methods

### 2.1. Study Design

The objective of this study was to identify and map how occupational therapy–related assessments and interventions have been reported in prospective interventional studies and case reports on DRFs, including studies addressing complications, and to identify literature gaps for future occupational therapy research. There are various types of literature reviews. Generally, when the feasibility, appropriateness, importance, or effectiveness of therapeutic and medical interventions is uncertain, a systematic review may be considered appropriate [24]. A scoping review is a type of literature review. According to Arksey and O′Malley, scoping reviews involve the following: (1) investigating the breadth, scope, and characteristics of research activities, (2) determining whether a systematic review is warranted, (3) summarizing and disseminating research findings, and (4) identifying research gaps in existing knowledge to comprehensively survey ongoing research and provide an overview of areas not yet explored [22]. We, therefore, conducted a scoping review to explore occupational therapy–related assessments and interventions for DRFs, including studies addressing complications. Articles were selected following a protocol developed in accordance with the Preferred Reporting Items for Systematic Reviews and Meta‐Analyses Extension for Scoping Reviews (PRISMA‐ScR); the protocol was established prior to study initiation [25].

### 2.2. Inclusion and Exclusion Criteria

The inclusion and exclusion criteria were developed using the Population–Concept–Context (PCC) framework, as recommended by the Joanna Briggs Institute for scoping reviews [26]. In the present study, PCC comprised the following: Population—adult patients aged ≥ 18 years with DRFs; Concept—occupational therapy–related assessments and interventions, regardless of inpatient or outpatient setting; and Context—English‐language articles. The eligibility criteria were as follows: (1) articles related to occupational therapy for DRFs, (2) studies involving adults aged ≥ 18 years (e.g., older adults), and (3) prospective interventional studies or case reports published as peer‐reviewed articles. The exclusion criteria were as follows: (1) articles on the development of scales or intervention methods, (2) articles evaluating the internal validity of assessments, (3) articles involving both physical therapy and speech therapy, (4) pediatric studies, and 5) retrospective or basic anatomical studies.

This study is also aimed at investigating the content of interventions. In a typical scoping review, grey literature is included; however, considering the objectives of this study, conference proceedings were excluded.

### 2.3. Search Strategy

The search strategy employed in this study involved using various literature databases, including PubMed, ProQuest, MEDLINE, OTseeker, and Cochrane Library. The last search date was January 31, 2025. The search terms were designed to capture as much relevant literature as possible related to DRFs and their complications. The target diagnoses included in the search formula were DRF, wrist fracture, Smith fracture, and Colles fracture. The search formula was as follows: “Distal Radius Fracture”[Text Word] OR “Radius Fracture”[Text Word] OR “Wrist Injuries”[Text Word] OR “Wrist Fractures”[Text Word] OR “Colles Fracture”[Text Word] OR “Smith Fracture”[Text Word]. Additionally, a search formula was used to comprehensively survey occupational therapy using the terms “Occupational Therapy”[Text Word] and “Hand Therapy”.

### 2.4. Data Charting

In the initial screening, the literature selection method involved six researchers independently reviewing the titles and abstracts of the searched articles to determine their eligibility. The literature data were managed using Microsoft Excel (Microsoft Corporation, Redmond, Washington, United States). In this study, data were sent to each researcher in an Excel file, and each researcher reviewed the data according to the eligibility criteria. When the opinion differed among researchers, they discussed among themselves to determine whether the abstract and literature should be included. After a decision was made regarding the abstract, the main text was evaluated by the researchers to determine whether it met the eligibility criteria. During the full‐text review, the same procedure used in the title and abstract screening was applied. All reviewers independently assessed the full texts against the eligibility criteria. Any disagreements were resolved through discussion among the reviewers until consensus was reached. The review team consisted of six researchers with backgrounds in occupational therapy, including clinicians and academic researchers with experience in rehabilitation, hand therapy, musculoskeletal conditions, and occupational therapy research. These professional backgrounds may have informed the interpretation of occupational therapy–related assessments and interventions. To enhance transparency and reduce potential bias, title and abstract screening, full‐text review, and data extraction were conducted independently by multiple reviewers, and disagreements were resolved through discussion until consensus was reached.

### 2.5. Data Extraction

The following data were extracted from the main text of included articles: year, country, research design, number of study participants, research facility, treatment method for the target diagnosis (conservative treatment, postsurgery, or both), inclusion and exclusion criteria, intervention content (intervention strategies and techniques), and results. Additionally, information on the evaluation items, assessment time, intervention period, end time, number of occupational therapy sessions, duration, and intervention content was extracted from the main text. The researchers then confirmed the extracted results, and any discrepancies were discussed among the researchers until consensus was reached. A critical appraisal of the methodological quality of the included studies was not conducted. This decision was made because the purpose of this scoping review was to identify and map the range, characteristics, and gaps in occupational therapy–related assessments and interventions for DRFs, rather than to evaluate the effectiveness of specific interventions or to synthesize evidence based on study quality. This approach is consistent with the purpose of scoping reviews, which generally aim to map the extent and nature of available evidence.

## 3. Results

### 3.1. Trial Flow (Figure 1)

**Figure 1:**
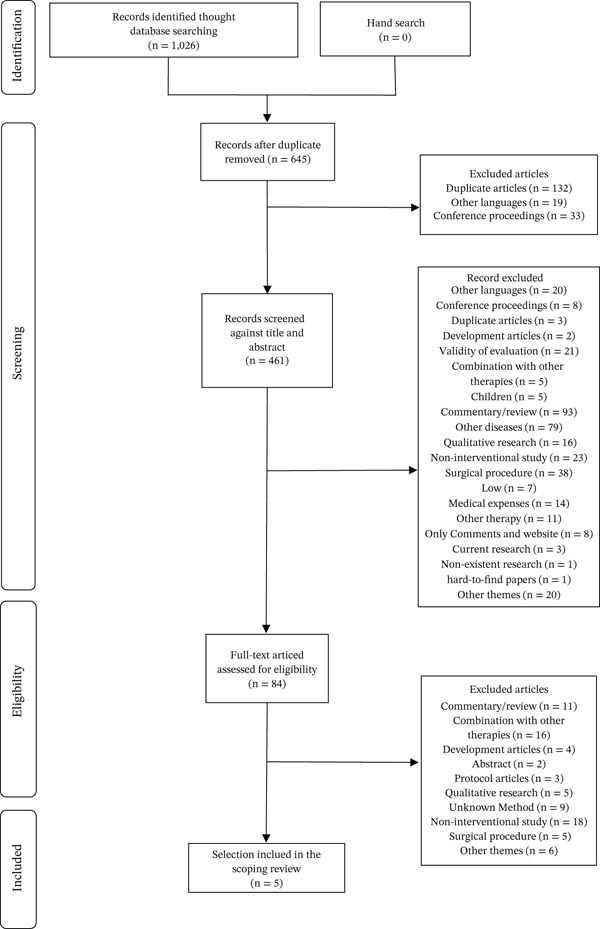
PRISMA‐ScR flow diagram showing the study selection process.

Figure 1 shows the PRISMA‐ScR flow diagram of the study selection process. The database search yielded 1026 articles. Duplicate articles were excluded, and the titles, abstracts, and main texts were carefully read, after which five research articles that met the inclusion criteria were finally selected [27–31].

### 3.2. Article Characteristics

Table 1 summarizes the characteristics of the articles: year, country, number of participants, research design, and implementing facility. All articles were prospective studies, with four being randomized studies [27, 29–31], while one article [28] was a nonrandomized interventional study.

**Table 1:** Characteristics of the included articles.

Article authors	Year	Country	Author	Number of subjects	Therapy	Study design	Implementing facility	Presence of complications	Selection criteria	Exclusion criteria
Kuo et al. (2013) [30]	2013	Taiwan	OT	22	Postoperative	Randomized controlled trial	Hospital	No	Age ≥ 50 years, no history of hand or forearm injury, no rheumatoid arthritis or osteoarthritis of the hand, and no other nerve disorders or severe soft tissue disorders	No description
Jarus et al. (2000) [28]	1999	Israel	OT	47	Both (no detailed description)	Interventional study	Clinic	No description	Traumatic fracture in one wrist, rehabilitation stage, wrist movement restriction, and edema compared to the uninjured hand	No description
Christensen et al. (2001) [27]	2001	Denmark	MD	30	No description	Randomized trial	Hospital	No description	No description	No description
Knygsand‐Roenhoej & Maribo (2011) [29]	2011	Denmark	OT	29	Both (11 conservative cases, 9 internal fixation cases, and 9 external fixation cases)	Randomized trial	Hospital	Yes	Age ≥ 18 years, unilateral distal radius fracture with plaster cast fixation, internal/external fixation, subacute edema, and volume difference between of UE ≥ 60 mL	Unable to participate due to mental illness, infection, visceral disease, and lymphedema
Souer et al. (2011) [31]	2011	United States	MD	94	Postoperative	Randomized controlled trial	Hospital	Yes	Age ≥ 18 years, surgery within 4 weeks after surgery, and fixation with locking plate and screws only	Other injuries, distal radius fracture with secondary fixation, and participant dependency

Abbreviations: CRPS, complex regional pain syndrome; MD, medical doctor; OT, occupational therapist; UE, upper extremity.

### 3.3. Characteristics of the Included Articles (Table 1)

Two articles targeted only postsurgery cases [30, 31], two articles targeted both postsurgery and conservative treatment cases [28, 29], and one article did not specify the treatment method [27]. The inclusion and exclusion criteria were as follows: Three articles applied age criteria [29–31], three articles applied a unilateral injury criterion [28, 29, 31], and one article did not specify any inclusion or exclusion criteria [27]. Two articles reported complications at the end of the intervention. Knygsand‐Roenhoej and Maribo reported one case each of CRPS and shoulder periarthritis in the intervention group [29], while Souer et al. reported eight cases of carpal tunnel release, three cases of tendonitis, and one case each of extensor pollicis longus tendon rupture and lunate alignment loss in the occupational therapy group, along with eight cases of carpal tunnel release in the control group [31].

### 3.4. Evaluation Period and Outcome Measures (Table 2)

**Table 2:** Evaluation period and outcome measures.

Article authors	Evaluation period	Range of motion	Grip strength	Pinch strength	Purdue Pegboard Test	Edema	Visual analog scale (pain)	PCS	Activities of daily living	DASH	COPM	Other
Kuo et al. (2013) [30]	Intervention group: 1 week after surgery and 3 weeks after surgery Control group: 1 week after removal of fixation and 12 weeks after surgery	◯	×	◯	◯	×	×	×	◯ (MAM‐36)	×	×	
Jarus et al. (2000) [28]	Total of six measurements: At the start of OT and once per week during therapy	◯	◯	×	×	◯	×	×	×	×	×	Surveys regarding interest and awareness of therapy
Christensen et al. (2001) [27]	3 months, 5 months, and 9 months	×	◯	×	×	×	×	×	×	×	×	
Knygsand‐Roenhoej & Maribo (2011) [29]	6 weeks and 9 weeks	◯	×	×	×	◯	◯	×	◯ (QBA)	×	◯	
Souer et al. (2011) [31]	3 months and 6 months	◯	◯	×	×	×	◯	◯	×	◯	×	PASS and CES‐D

Abbreviations: CES‐D, Center for Epidemiologic Studies Depression Scale; COPM, Canadian Occupational Performance Measure; DASH, Disabilities of the Arm, Shoulder and Hand; MAM‐36, Manual Ability Measure‐36; PCS, Pain Catastrophizing Scale; PASS, Pain Anxiety Symptoms Scale; QBA, questionnaire for bilateral activities.

Table 2 presents the details of the evaluation timing and items. The duration of evaluation varied among studies, but three articles included baseline measurements at the start of the intervention and 12 weeks postintervention [27, 30, 31]. The evaluation items used in the studies were as follows: Four articles used joint range of motion measurements [28–31], and three articles used grip strength measurements [27, 28, 31]. For pain‐related evaluations, two articles used the Visual Analog Scale [29, 31], and one article used the Pain Catastrophizing Scale [31]. Other evaluation items were as follows: Two articles evaluated activities of daily living (ADLs) [29, 30], one article used the Disabilities of the Arm, Shoulder and Hand [31], and one article used the COPM [29].

### 3.5. Characteristics of the Interventions (Table 3)

**Table 3:** Characteristics of the interventions.

Article authors	Start period	Intervention end period	Number of sessions	Time	Presence of external fixation	Intervention content (Intervention group)	Intervention content (Control group)
Kuo et al. (2013) [30]	First week after surgery	12 weeks	3 times a week	45 min per session	Yes	Implementer: Occupational therapist Weeks 1–2: Massage, passive ROM training, stretching, active ROM training of the affected joint, and wound care Weeks 3–4: Massage, active ROM training of affected joint, stretching, ADL training, and pain‐free isometric exercises of fingers Weeks 5–6: Massage, active ROM training of affected joint, stretching, ADL training, pain‐free isometric exercises of fingers, and finger tendon gliding exercise Week 7 (fixation device removal): Wound care, active ROM training of the wrist, and ADL training Weeks 8–9: Wound care, active or passive ROM training of wrist, ADL training, wrist mobilization, and wrist strengthening Weeks 10–12: Wound care, active or passive ROM training of wrist, ADL training, wrist mobilization, wrist muscle strengthening, and hardening training	Implementer: Unknown Weeks 1–2: Elevation of the upper limb and active ROM training of the shoulder joint and the elbow joint Weeks 3–6: Elevation of upper limb, active ROM training without pain, and passive ROM training of shoulder joint, elbow joint, and wrist joint Weeks 1–6: External fixation conducted and only instructions given After Week 7, rehabilitation was conducted using the same method as the intervention group
Jarus et al. (2000) [28]	None	5 weeks	3 times a week	50 min per session	No	Implementer: Occupational therapist Method: 10 min in whirlpool, 10 min of massage and passive exercises, 10 min of training with therapist, computer use for 10 min, and pronation, extension, flexion, and extension for 10 min	Implementer: Occupational therapist Method: 10 min in whirlpool, 10 min of massage and passive exercises, 10 min of training with therapist, brush machine use for 10 min, and pronation, extension, flexion, and extension for 10 min
Christensen et al. (2001) [27]	After cast removal	No description	3 times a day	No description	Yes	Implementer: Occupational therapist Method: Active joint movement of wrist, elbow, and shoulder joints, edema prevention, conditioning training, gross and fine motor training, muscle strengthening training, sensory training, and ADL training	Implementer: Unknown Method: 10 min of warm‐up with warm soapy water; ROM training of shoulder, wrist, and finger joints; and exercise for lower back movement
Knygsand‐Roenhoej & Maribo (2011) [29]	None	Up to 9 weeks	3 times a week for 4 weeks, then 2 times a week	No description	No	Implementer: Occupational therapist Method: Edema therapy, ROM training, muscle strengthening training, modified MEM used for edema therapy, and Isotoner open‐finger gloves and multilayer bandages prescribed	Implementer: Occupational therapist Method: Edema therapy, ROM training, muscle strengthening conditioning, occupational therapy, and the use of Isotoner open‐finger gloves at night
Souer et al. (2011) [31]	None	None	Intervention: 3–4 times a day Control: Determined by OT	Intervention: 30 min per day Control: Determined by OT	Yes	Implementer: Occupational therapist Method: Only the occupational therapy group is described	Implementer: Surgeon The following procedures were performed by the surgeons, not by therapists. Method: Finger flexion and extension, elbow extension, shoulder joint abduction to maintain horizontal orientation, active joint movement, and wide wrist movement using the other arm

Abbreviations: ADL, activity of daily living; MEM, manual edema mobilization; OT, occupational therapist; ROM, range of motion.

Table 3 provides details regarding the intervention period, number of sessions, and method of occupational therapy used. One article explicitly stated the intervention start and end periods [30]. The contents of the occupational therapy interventions were as follows: Four articles included a protocol [27–30], two of which incorporated strength training and ADL training [27, 30]. However, detailed descriptions of the ADL training were not provided in either study.

## 4. Discussion

In this scoping review, we aimed to identify and map occupational therapy–related assessments and interventions reported in prospective interventional studies and case reports on DRFs, including studies addressing complications. The results revealed that only five studies met the inclusion criteria, and the included studies generally employed relatively narrow eligibility criteria. This finding is consistent with those of Trzeciak and Małek, who reported that occupational therapy studies on DRFs often excluded patients with complications, thereby limiting the study population [18]. In the present review, narrow eligibility criteria refer to the exclusion of patients with additional injuries, complex clinical presentations, or other conditions that may influence intervention outcomes. Such criteria may limit the generalizability of findings to patients with DRFs encountered in routine clinical practice, including those with complications. Herein, we discuss the findings of the evaluations and interventions used in the included studies. Specifically, this review is aimed at identifying how occupational therapy–related assessments and interventions have been reported in prospective interventional studies and case reports on DRFs, including studies addressing complications, and at clarifying literature gaps for future occupational therapy research.

### 4.1. Evaluations and Interventions

Review results showed that 80% of the articles used range of motion and grip strength measurements as evaluation items. Goldhahn et al. reported that approximately 50% of DRF studies used range of motion or grip strength as outcome measures [32]. In the present review, the proportion was higher, with 80% of included studies using these measures. This finding is consistent with previous research indicating that impairment‐level outcomes are commonly used in DRF studies. As background factors, outcome assessment after DRFs includes not only radiographic evaluation but also range of motion, grip strength, pinch strength, and patient‐reported functional outcome measures [33, 34]. Among these, wrist range of motion is the most frequently reported outcome measure following DRFs. Furthermore, because the present study focused on interventional studies, these factors may explain the results observed in this study. These findings suggest that occupational therapy–related research on DRFs has primarily emphasized body functions and structures, such as wrist motion and hand strength, rather than activity, participation, or occupational performance.

Additionally, range of motion and grip strength measurements were used as indicators of fracture treatment and healing [35]. Therefore, these measures may have been frequently selected in the included studies because they are familiar, feasible, and directly related to physical recovery after DRFs.

In this study, the COPM was used in only a small number of studies. The limited use of the COPM suggests that client‐identified occupational performance and satisfaction have not been sufficiently examined in DRF intervention research. A previous [36] scoping review focused on the functional recovery of DRFs without complications and reported that approximately 60% of the articles evaluated range of motion, while approximately 10% used the COPM. These findings suggest that occupational therapy assessments addressing activity and participation have been used only to a limited extent in research on DRFs. The COPM is an evaluation tool based on the Canadian Model of Occupational Performance and Engagement [17, 37, 38].

Furthermore, the COPM is an evaluation method that considers changes in clients′ perceptions regarding occupations and emphasizes activity and participation. Because the COPM captures activity and participation from the client′s perspective, its use may help identify occupational challenges that are not fully reflected by impairment‐level outcomes such as range of motion, grip strength, pain, or edema. Occupational therapists are professionals who listen to the needs of clients and conduct evaluations and interventions that focus on activity and participation. In contrast, in the field of DRFs, research appears to have predominantly focused on physical functions, with comparatively limited attention to occupation‐focused approaches.

Among the included studies, the study by Kuo et al. [30] was the only study that described the intervention content in detail, covering range of motion training, muscle strength training, and ADL, whereas most studies focused primarily on range of motion training. Although ADL training was reported in some intervention protocols, the content was not described in sufficient detail to determine whether these interventions were genuinely occupation‐focused or primarily impairment‐oriented exercises integrated into daily activities. Based on the findings of the present study and previous research, many studies conducted by occupational therapists have tended to focus on physical functions, such as range of motion and grip strength, rather than on occupation‐focused interventions. Although occupational therapists are professionals who listen to clients′ needs and focus on activity and participation, the findings of the present review suggest that such occupation‐focused perspectives have been insufficiently reflected in research on DRFs. Another important consideration is that DRF rehabilitation is frequently provided within hand therapy settings, where occupational therapists and physical therapists may have overlapping roles. Therefore, occupational therapy–related interventions may not always be clearly distinguished from broader hand therapy interventions in the literature. This may partly explain why many studies emphasize biomechanical outcomes and treatment protocols rather than occupational performance or participation.

Nielsen and Dekkers reported that patients with DRFs continued to experience challenges with issues such as self‐care even 1 year later and emphasized the importance of long‐term interventions from the perspective of occupational therapy [39]. Therefore, conducting research that focuses on activities and participation in daily life, such as self‐care, will contribute to establishing evidence on this topic.

### 4.2. Areas for Future Research

The results of our study revealed that research on occupational therapy, including complications, comprised only five interventional studies. When compared to previous research, a systematic review by Pradhan et al. on the effects of rehabilitation for patients with wrist fractures reported that two out of 21 studies incorporated occupational therapy [40], suggesting a tendency for fewer studies to include intervention by occupational therapists. Additionally, as reported by Trzeciak and Małek [18], research on occupational therapy for DRFs excluded cases with complications and limited the selection of participants. One reason for this was the issue of participant recruitment. In previous research, a literature review demonstrated a reluctance to recruit patients with greater functional limitations [41]. Patients with complications of DRFs are more likely to be patients with greater functional limitations compared to those with simple fractures, and there would be barriers to their recruitment. As such, the applicability of the results to the general patient population with fractures is limited. Furthermore, research in the field of CRPS has shown that, at 12 months, grip strength is reduced by approximately 25%–66%, and joint range of motion is decreased by about 20%–25%. In addition, approximately 30%–40% of patients are unable to return to work, and a further 27%–35% are able to return to work only with some form of workplace accommodation [42]. These findings suggest that CRPS affects not only physical function but may also have a substantial impact on activities and participation.

Furthermore, the aim of these studies may have been to identify significant differences [43, 44]. Clinical trials are typically categorized into pragmatic and explanatory trials, with explanatory trials focusing on the effectiveness of the intervention and controlling various factors that may impact the evaluation, such as the participants [44, 45]. Although clinical trials should have sufficiently broad eligibility criteria to ensure generalizability to the general population [43], limiting participant eligibility and prioritizing intervention efficacy may hinder the verification of treatment effectiveness in a wider patient population [43, 45]. Future research should include not only explanatory designs with relatively homogeneous samples, when appropriate, but also pragmatic designs with broader eligibility criteria that allow the inclusion of patients with complications, thereby improving generalizability to routine clinical practice [43, 45, 46].

From the perspective of the ICF, the findings of this review indicate an imbalance between outcomes related to body functions and structures and those related to activity and participation. Moreover, results of our study indicate that only two out of five articles evaluated activity and participation within the ICF framework [29, 30], and a diverse range of evaluation parameters were used. The report by Schoneveld et al. [47] presented five types of assessments for activity and participation in hand injuries, but few were utilized from the perspective of the ICF. The combination of two types of assessments was recommended when evaluating activity and participation. Therefore, the field of DRFs as a whole tends to focus on improving clinical outcomes such as physical function, and the results of our study reflect the problem of the lack of evaluation scales for activity and participation.

Additionally, the historical background may have influenced the predominance of research focusing on body functions and structures. In the field of occupational therapy, a considerable paradigm shift referred to as the mechanistic paradigm occurred from the 19th to the early 20th century. This shift resulted in the development of biomechanical models centered on anatomy and motion dynamics, with occupational therapy interventions targeting conditions such as musculoskeletal injuries [38]. This also led to an increase in research focusing on mental and physical functions, as well as body structure. However, Wong and Fisher noted that occupational therapists working in hand therapy may prioritize the use of a biomechanical framework as the primary model and an occupation‐focused model as complementary [37]. Furthermore, they stated that therapists may not rely on models at the initial stage of therapy to address comprehensive occupational goals and problems but may instead combine client‐centered goals and interests, models addressing learning and motivation in the context of occupation, and occupation‐focused models [37].

Recent reports have emphasized the need for appropriate occupational therapy interventions for CRPS, a common complication of DRFs [48, 49]. Because CRPS may affect not only pain, range of motion, and grip strength but also return to work, daily activities, and participation, impairment‐level outcomes alone may be insufficient to capture the full impact of this complication. Therefore, in such cases, evaluations and interventions focusing on activity and participation, rather than solely on impairment‐based improvement, are considered essential.

It is anticipated that, by adopting assessments grounded in the ICF framework and approaches that focus on occupation, challenges related to activity and participation—beyond mental and physical functions and body structure—can be more clearly identified. Furthermore, employing occupation‐focused models that emphasize activity and participation may help verify the effects of rehabilitation and occupational therapy for individuals experiencing complex occupational challenges, such as those associated with CRPS.

## 5. Limitations

Our study is a review that is limited to prospective interventional studies and does not represent all research on DRFs. Our study is also limited to English‐language literature and does not reflect specific data and findings on occupational therapy, making it challenging to fully apply our results to clinical practice or research fields. Furthermore, Valdes et al. reported that, in the field of hand therapy and rehabilitation for DRFs, interventions may be provided by physical therapists in addition to occupational therapists, and surgeons may also provide instruction in home programs [50]. Therefore, because the present review focused solely on occupational therapy–related studies, research findings from the broader hand therapy literature may not have been fully captured.

## 6. Conclusions

Our study focused on occupational therapy research for DRFs, including cases with complications. First, we investigated and summarized the content of occupational therapy evaluations and interventions for DRFs, regardless of complications. We subsequently conducted a literature review using a scoping review method, aiming to summarize research on occupational therapy in this field and identify the factors necessary for future research. Our findings indicate that occupational therapy–related studies on DRFs have primarily used impairment‐level outcomes and biomechanical interventions, such as range of motion and grip strength, whereas activity, participation, occupational performance, and psychosocial aspects have been less frequently addressed. The small number of included studies and their relatively narrow eligibility criteria suggest that existing evidence may have limited applicability to the broader population of patients with DRFs, particularly those with complications. Therefore, future research should place greater emphasis on occupation‐focused perspectives. Furthermore, conducting research that includes cases with complications in the study population and developing a research design that aligns with daily clinical practice will help verify the effectiveness of rehabilitation and occupational therapy for DRFs.

## Author Contributions

Junichiro Muranaka, Yuko Shigeta, Kohei Ikeda, Masatoshi Gocho, and Satoshi Sasada are co‐authors.

## Funding

No funding was received for this manuscript.

## Conflicts of Interest

The authors declare no conflicts of interest.

## General Statement

*Selected Publication.* Okita Y, Kawaguchi Y, Inoue Y, et al. Characteristics of goal‐setting tools in adult rehabilitation: A scoping review. *Clinical Rehabilitation*. 2023; 38(2): 234‐250. doi:10.1177/02692155231197383. *Institutional Profile*. http://www.kuhs.ac.jp/department/graduate_school/professors/details_01435.html?l=professors_rehabilitation_ot.

## Data Availability

Data available on request from the authors. The data that support the findings of this study are available from the corresponding author upon reasonable request.
